# Nimodipine Exerts Time-Dependent Neuroprotective Effect after Excitotoxical Damage in Organotypic Slice Cultures

**DOI:** 10.3390/ijms23063331

**Published:** 2022-03-19

**Authors:** Urszula Hohmann, Chalid Ghadban, Tim Hohmann, Joshua Kleine, Miriam Schmidt, Christian Scheller, Christian Strauss, Faramarz Dehghani

**Affiliations:** 1 Medical Faculty, Institute of Anatomy and Cell Biology, Martin Luther University Halle-Wittenberg, 06112 Halle (Saale), Germany; urszula.hohmann@medizin.uni-halle.de (U.H.); chalid.ghadban@medizin.uni-halle.de (C.G.); tim.hohmann@medizin.uni-halle.de (T.H.); joshua.kleine@student.uni-halle.de (J.K.); miriam.schmidt@student.uni-halle.de (M.S.); 2 Department of Neurosurgery, Medical Faculty, Martin Luther University Halle-Wittenberg, 06120 Halle (Saale), Germany; christian.scheller@klinikum-bremerhaven.de (C.S.); christian.strauss@uk-halle.de (C.S.)

**Keywords:** nimodipine, excitotoxicity, nifedipine, neuroprotection, microglia

## Abstract

During injuries in the central nervous system, intrinsic protective processes become activated. However, cellular reactions, especially those of glia cells, are frequently unsatisfactory, and further exogenous protective mechanisms are necessary. Nimodipine, a lipophilic L-type calcium channel blocking agent is clinically used in the treatment of aneurysmal subarachnoid haemorrhage with neuroprotective effects in different models. Direct effects of nimodipine on neurons amongst others were observed in the hippocampus as well as its influence on both microglia and astrocytes. Earlier studies proposed that nimodipine protective actions occur not only via calcium channel-mediated vasodilatation but also via further time-dependent mechanisms. In this study, the effect of nimodipine application was investigated in different time frames on neuronal damage in excitotoxically lesioned organotypic hippocampal slice cultures. Nimodipine, but not nifedipine if pre-incubated for 4 h or co-applied with NMDA, was protective, indicating time dependency. Since blood vessels play no significant role in our model, intrinsic brain cell-dependent mechanisms seems to strongly be involved. We also examined the effect of nimodipine and nifedipine on microglia survival. Nimodipine seem to be a promising agent to reduce secondary damage and reduce excitotoxic damage.

## 1. Introduction

Nimodipine has been found to be beneficial in many central nervous system disorders, including stroke, brain injury, cerebral ischemia, epilepsy, dementia and age-related degenerative diseases [1,2,3,4]. Nimodipine acts as a potent cerebral vasodilator and binds to cell membranes (K_D_(human) = 0.27 nM) and is a more lipophilic molecule than the calcium channel antagonist nifedipine [5]. Furthermore, the tissue concentration after application is three times higher than for nifedipine, indicating differences in crossing the blood–brain barrier [5]. Clinical and in vivo studies with nimodipine have demonstrated protection against ischemic damage [6] and an increased postischemic perfusion. Although this is a possible vascular mechanism for nimodipine’s protective effect, an additional direct effect by blocking calcium entry into neurons has also been suggested. In animal models, nimodipine has been shown to induce neuroprotection against glutamate or amyloid β-induced toxicity [7] and has been found to improve dementia [8] and memory in a variety of cognitive tests in aging subjects [9]. Nimodipine has affected neurons in the hippocampus in different studies [2,5,9,10,11,12,13,14,15]. Effects in the hippocampus seem to be independent from the vasculature [10]. In most in vitro studies, the effective concentrations of nimodipine were considerably higher than required for its cerebro-vascular effects; for this reason, nimodipine at therapeutic doses seems not to affect the release of neurotransmitters from neurons in healthy brain tissue [5]. 

On the cellular level, both microglia and astrocytes were influenced by nimodipine [16,17,18]. The neuroprotective effect of nimodipine in inflammation-mediated neurodegenerative disease was attributed to the inhibition of microglial activation, since nimodipine significantly inhibited the production of nitric oxide (NO) and further cytokines from lipopolysaccharide (LPS)-stimulated cells [16]. Activated microglia migrate to the lesion site and proliferate [19]. Changes in the number of microglia are associated with neuronal damage. However, both increases in the pro-reparative and decreases in the pro-inflammatory population might induce neuroprotective effects [19]. Furthermore, nimodipine attenuated neurotoxicity induced by interferon (IFN) γ in human astrocytes [17]. 

Nifedipine, a further calcium antagonist, also displayed neuroprotection but at a lower potency and in much higher concentrations (100 μM) than nimodipine [20]. Nimodipine was neuroprotective, whereas nifedipine exerted no effects in clinical applications and in in vitro models [5,21]. 

In this study, organotypic hippocampal slice cultures (OHSC), a well-studied model with physiological neuronal cell morphology, and their in vivo-like organization were used to further characterize direct cellular effects of nimodipine [22].

Since nimodipine was neuroprotective against excitotoxical damage in cell culture [11,12], an influence of nimodipine and nifedipine on a neuronal damage model of excitotoxically lesioned OHSC was examined. Furthermore, the effect of calcium antagonists on microglia viability was assessed.

## 2. Results

### 2.1. Nimodipine Is Protective When Administered Simultaneously with NMDA

**Figure 1:**
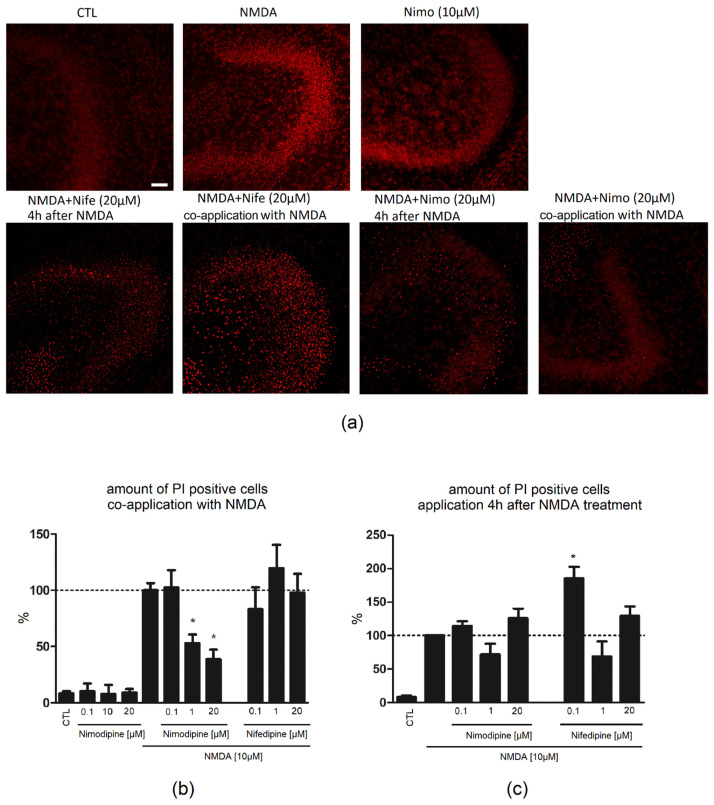
Effect of nimodipine and nifedipine after NMDA damage at different time points of application. Amount of PI-positive damaged neurons after NMDA (10 µM) damage followed by treatment with nimodipine (0.1, 1, 20 µM) or nifedipine (0.1, 1, 20 µM). (**a**) In CTL slices, few PI-positive cells (red) were found, whereas after NMDA treatment, a massive increase in the number of dead cells in GCL of DG was observed. Representative pictures for nimodipine (20 µM) and nifedipine (20 µM)-treated slices after/during neuronal damage (**b**) In the control group, few positive neurons were detected (n_CTL_ = 30). Incubation with NMDA (n_NMDA_ = 20) over 4 h induced a massive increase in the number of damaged cells in the region of interest. Nimodipine, when applied alone to OHSC had no significant effect on the number of PI-positive damaged cells (n_0.1µM_ = 6; n_10µM_ = 3; n_20µM_ = 9). Nimodipine (n_0.1µM_ = 5; n_1µM_ = 15; n_20µM_ = 19) but not nifedipine (n_0.1µM_ = 11; n_1µM_ = 10; n_20µM_ = 7) was protective after NMDA (10 µM) lesion, when applied directly with NMDA. (**c**) Nimodipine (n_0.1µM-4 h_ = 4; n_1µM-4 h_ = 8 n_20µM-4 h_ = 16) and nifedipine (n_0.1µM-4 h_ = 3, n_1µM-4 h_ = 4; n_20µM-4 h_ = 11) applied after 4 h showed no protective effects after NMDA damage in OHSC. All values were normalized to NMDA. Data are presented as mean with SEM. * depict statistically significant results with *p* < 0.05. Scale bar = 50 µM.

The number of Propidium Iodide (PI)-positive neurons was assessed, and all data were normalized to the N-Methyl-D-Aspartat (NMDA, 10 µM) group. Control slices (CTL) exhibited a good neuronal preservation. Only few PI-positive nuclei were found in the granule cell layer (GCL) of the dentate gyrus (DG) (8.43%/GCL, Figure 1b). Nimodipine (0.1 µM: 10.35%/GCL; 10 µM: 7.96%/GCL; 20 µM: 9.21%/GCL, Figure 1a) alone had no significant effect on the number of PI-positive cells in the GCL. 

Lesion of OHSC with 10 µM NMDA resulted in a massive accumulation of PI-positive nuclei in the DG (100%/GCL, Figure 1a,b). Treatment of lesioned OHSC with nimodipine 0.1 µM (102.4%/GCL) parallel to NMDA had no effect on cell degeneration, whereas a higher concentration (1 µM: 52.88%/GCL; 20 µM: 38.83%/GCL) led to a significant reduction in the number of PI-positive cells. Parallel incubation with nifedipine did not influence (0.1 µM: 83.28%/GCL; 1 µM: 119.7%/GCL; 20 µM: 98%/GCL) the neuronal damage. 

**Figure 2:**
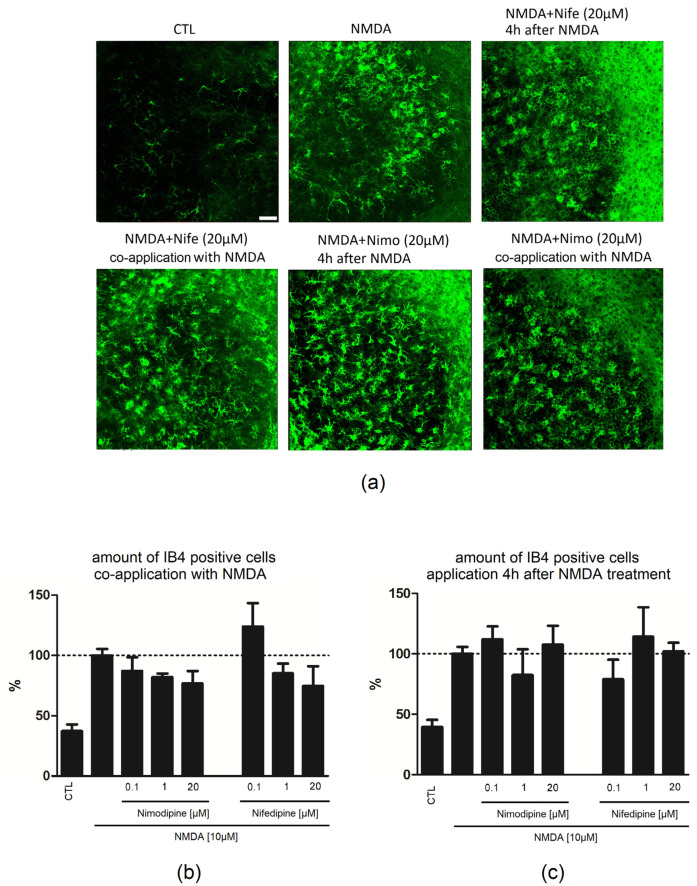
Effects of nimodipine or nifedipine after NMDA damage at different time points of application. Amount of IB4-positive microglia cells after NMDA (10 µM) lesion followed by treatment with nimodipine (0.1, 1, 20 µM) or nifedipine (0.1, 1, 20 µM). (**a**) In CTL slices, few IB4-positive cells (green) were found, whereas NMDA treatment led to a massive increase in the number of IB4-positive cells in GCL of DG. Representative pictures for nimodipine (20 µM) and nifedepine (20 µM)-treated slices after/during neuronal damage (**b**) In the control group, few IB4-positive cells were detected (n_CTL_ = 21). Incubation with NMDA (n_NMDA_ = 35) over 4 h induced a massive increase in the number of IB4-positive cells in the DG. Nimodipine (n_0.1µM_ = 5; n_1µM_ = 3; n_20µM_ = 10) and nifedipine (n_0.1µM_ = 5 n_1µM_ = 6; n_20µM_ = 7) did not significantly affect the number of microglia after NMDA (10 µM) lesion, when applied directly with NMDA. (**c**) Nimodipine (n_0.1µM-4 h_ = 4; n_1µM-4 h_ = 4; n_20µM-4 h_ = 10) and nifedipine (n_0.1µM-4 h_ = 9, n_1µM-4 h_ = 4; n_20µM-4 h_ = 14), when applied 4 h after the induction of injury, showed no significant effects on the number of IB4-positive cells. All values were normalized to those of the NMDA group. Data are presented as mean with SEM. Scale bar = 50 µM.

Application of NMDA (100%/GCL) led to an increase in the number of IB4-positive microglia in comparison to CTL (37.27%/GCL). Combined treatment of nimodipine (0.1 µM: 87.12%/GCL; 1 µM: 82.08%/GCL; 20 µM: 76.67%/GCL) or nifedipine (0.1 µM: 123.9%/GCL; 1 µM: 85.22%/GCL; 20 µM: 74.70%/GCL) with NMDA resulted in no significant changes in the number of IB4-positive cells (Figure 2).

### 2.2. Nimodipine Showed No Neuroprotective Effects When Applied after Neuronal Damage

Application of nimodipine (0.1 µM: 114.3%/GCL; 1 µM: 71.80%/GCL; 20 µM: 125.8%/GCL) or nifedipine (0.1 µM: 185.5%/GCL; 1 µM: 68.92%/GCL; 20 µM: 129.2%/GCL) 4 h after beginning of the lesion with NMDA did not lead to a reduction in the number of PI-positive cells (Figure 1c). No significant changes in the number of IB4-positive cells was observed when nimodipine (0.1 µM: 111.9/GCL; 1 µM: 82.29%/GCL; 20 µM: 107.5%/GCL) or nifedipine (0.1 µM: 78.76%/GCL; 1 µM: 114.1%/GCL; 20 µM: 102%/GCL) were applied 4 h after beginning of the lesion with NMDA (Figure 2).

### 2.3. Four Hour Preincubation with Nimodipine Is Protective, Whereas 24 h Preapplication of Nimodipine or Nifedipine Had No Effect on Neuronal Damage

**Figure 3:**
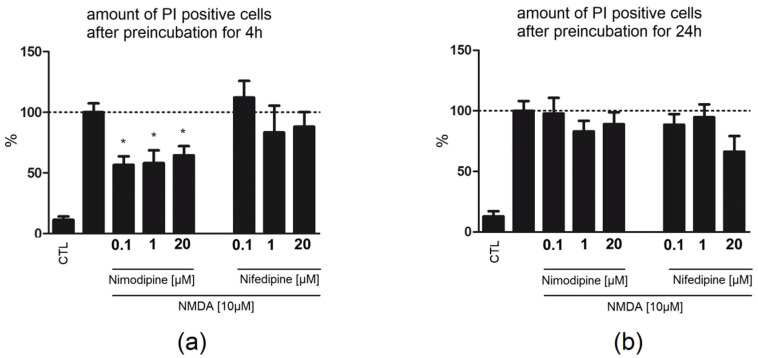
Effect of nimodipine and nifedipine in NMDA-damaged OHSC at different time points of application. Only few cells were positive in the control group ((**a**) n_CTL_ = 18; (**b**) n_CTL_ = 17). Application of NMDA ((**a**) n_NMDA_ = 28; (**b**) n_NMDA_ = 27) for 4 h led to accumulation of PI-positive nuclei in the dentate gyrus. (**a**) Preincubation (4 h) with nimodipine (n_0.1µM_ = 8; n_1µM_ = 11; n_20µM_ = 9) led to significant reduction in the number of damaged neurons in comparison to nifedipine treatment (n_0.1µM_ = 9; n_1µM_ = 9; n_20µM_ = 10) that showed no reduction (**b**) Nimodipine (n_0.1µM_ = 19; n_1µM_ = 16; n_20µM_ = 13) or nifedipine (n_0.1µM_ = 18; n_1µM_ = 12; n_20µM_ = 5), when pre-applied for 24 h before NMDA-damaged OHSC had no significant effect on the number of PI-positive damaged cells. All values were normalized to NMDA. Data are presented as mean with SEM. * depict statistically significant results with *p* < 0.05.

Application of nimodipine 4 h before NMDA damage until fixation was protective (0.1 µM: 56.6%/GCL; 1 µM: 58.1%/GCL; 20 µM: 64.38/GCL) in contrast to nifedipine (0.1 µM: 112.2%/GCL; 1 µM: 83.26%/GCL; 20 µM: 88.06%/GCL, Figure 3).

Preincubation for 24 h with nimodipine (0.1 µM: 97.82%/GCL; 1 µM: 83.01%/GCL; 20 µM: 89/GCL) or nifedipine (0.1 µM: 88.53%/GCL; 1 µM: 94.63%/GCL; 20 µM: 66.34%/GCL, Figure 3) for 24 h followed by co-application with NMDA and incubation after the lesion until fixation did not lead to reduction in the number of PI-positive cells.

### 2.4. Application of Nimodipine Had No Effect on Microglia Cell Death

**Figure 4:**
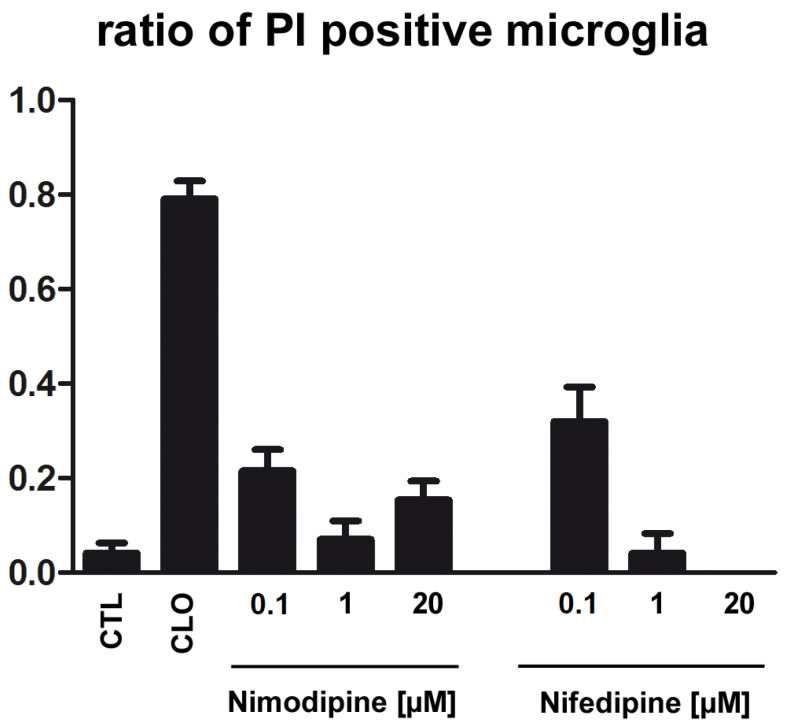
Effects of nimodipine or nifedipine on microglia cell death. The application of nimodipine (n_0.1µM_ = 21; n_1µM_ = 15; n_20µM_ = 17) or nifedipine (n_0.1µM_ = 13; n_1µM_ = 6; n_20µM_ = 7) had no effect on cell death in primary microglia (5000). Clodronate (n_CLO_ = 59) increased the number of damaged microglia in comparison to CTL (n_CTL_ = 25) with typical changes in morphology, whereas nimodipine or nifedipine had no effect on cell death. Data from three independent experiments are presented as mean with SEM.

Nimodipine (0.1 µM:0.21; 1 µM: 0.07; 20 µM: 0.15) or nifedipine (0.1 µM:0.32; 1 µM: 0.04; 20 µM: 0.0) displayed no statistically resolvable effect on microglial cell death in primary cell culture, whereas clodronate (CLO: 0.79) depleted microglia significantly in comparison to CTL (0.04) (Figure 4).

## 3. Discussion

Nimodipine, a 1,4-dihydropyridine Ca^2+^ channel antagonist, has been used as a neuroprotective agent in subarachnoidal haemorrhage (SAH) against vasospasm [23,24]. Improved clinical outcome of patients seems to be associated with additional Ca^2+^ channel independent mechanisms such as inhibition of vasospasm, increase in fibrinolytic activity, neuroprotection, reduction of spreading, depolarization and inhibition of microthromboembolism [6,25]. Furthermore, in various in vivo and in vitro models of cerebral ischemia, nimodipine was found to be protective [4,6]. 

### 3.1. Nimodipine but Not Nifedipine Is Protective in OHSC

Whereas the primary injury cannot be reversed, the secondary injury, as a result of destructive and self-propagating biological changes in cells and tissues leads to further dysfunction and cell death after hours to weeks [26]. The therapeutic actions focus on deceleration and containment of cellular and molecular mechanisms during the secondary injury [27]. However, effective drugs are missing. 

In this study, organotypic hippocampal slice cultures (OHSC), a well-studied model with unaltered morphology of neuronal cells and their in vivo-like organization, was used. 

In order to simulate neuronal damage, NMDA was applied to OHSC to induce excitotoxicity. Excitotoxicity is a complex process triggered by glutamate receptor activation that results in Ca^2+^ overload, which activates various intracellular mechanisms, enzymes and free radicals, leading to degeneration of dendrites and cell death [28]. It has recently become clear that there exists a number of subtypes of apoptosis and an overlap between apoptosis, necrosis and autophagy. Cells can die via different mechanisms with partially high mechanistic overlap and with some forms of induced cell death cascades being reversible, even in late stages [29]. Excitotoxic neuronal death as observed in OHSC is characterized by a continuum of necrotic, apoptotic, and autophagic events [30], which can be visualized by PI labelling [31]. The degradation of neurons is associated with an inflammatory response from glia cells and peripheral immune cells [27]. From a mechanistic point of view, calcium antagonists might be promising agents counteracting excitotoxicity. In our study, nimodipine but not nifedipine reduced the secondary damage in OHSC if co-applied with NMDA. Findings on nimodipine (2–5 µM) are in accordance with previous studies showing protective effects in hippocampal neurons against glutamate excitotoxicity [12,32,33]. While NMDA receptor activation is a primary contributor to excitotoxic injury, the relative contribution of voltage-dependent calcium channels to excitotoxicity may differ depending on particular type of neuron. An antagonist that selectively blocks one of the different glutamate receptors or Ca^2+^ channels may therefore exhibit differential effectiveness in protecting different populations of neurons [28]. Consistent with this idea, nimodipine at various concentrations was shown to protect against injury generated by exogenous application of NMDA or glutamate to cultured hippocampal neurons [11]. In the OHSC model, simultaneous application of nimodipine with damaging NMDA was protective in concentrations of 1 and 20 µM. Nifedipine showed no protective effect. It seems plausible that further unknown mechanisms next to calcium antagonisation exist for nimodipine-mediated neuroprotection that are absent for nifedipine. 

Several studies reported on reductions of L-type calcium currents by nimodipine in hippocampal CA1 neurons [2,13,14,34]. Furthermore, and in agreement with this study, investigations in in vitro and animal models indicated neuroprotection against glutamate or amyloid β-induced toxicity [7] and in focal ischemia (MCA occlusion) [5]. In mesencephalic neuron–glia cultures and in NGF-differentiated PC-12 cells, nimodipine had neuroprotective effects [16,20]. In line with our findings, Nuglisch and colleagues reported on the neuroprotective effect of nimodipine independent of cerebral vasodilation and suggested direct actions on neurons or glial cells [35]. Nimodipine (10 µM) was found to completely block synaptic activity, significantly reduce the toxicity induced by 0.1 mM magnesium, and protect hippocampal cultures from excitotoxicity [12]. Notably, higher nimodipine concentrations might express nonselective effects or inhibit further channels or targets [12]. In rat cortical synaptosomes, nimodipine at 0.5 to 25 µM inhibited the release of endogenous glutamate that was correlated with the inhibition of Ca^2+^ uptake [5]. 

In substantia nigra but not in the tegmental area, both nimodipine and nifedipine improved survival of dopaminergic neurons after 4 weeks of application [36]. Furthermore, nimodipine but not nifedipine ameliorated survival of Neuro2a cells [37], and nimodipine rescued Neuro2a cells from ethanol-, heat- and mechanically induced cell death in a dose-dependent manner [38]. In the majority of models, nimodipine but not nifedipine showed positive effects on neuronal survival.

Still, there is some controversy about nifedipine-mediated neuroprotection. Some authors observed for nifedipine protective effects after acute axotomy [36], in dopaminergic neurons [39] and in pancreatic β-cells [40,41]. In addition, nifedipine but not nimodipine was found to exhibit antioxidant properties [42,43]. In our model, nifedipine (0.1 µM) increased the number of damaged neurons if applied 4 h after neuronal damage, and there is no clear explanation for this effect. It was shown before that nifedipine in smaller doses was toxic in patients [44]. It was also shown that nifedipine alters lipid concentration, and that lipids are mediators of neurotoxic effects of astrocytes, which may be the mechanism behind nifedipine toxicity in this model. However, the mechanism behind nifedipine-mediated toxicity 4 h after NMDA damage is unclear [45,46]. Despite protective properties of nifedipine due to the blockade of calcium channels, nimodipine seems to involve additional and other mechanisms [5,6]. The data hint to a possible variable expression of targets between cell and tissue types; as well, the region of the central nervous system seems to be crucial. 

### 3.2. The Absence of Functional Blood Vessels in OHSC and the Neuroprotective Effect of Nimodipine but Not Nifedipine Strengthens the Presence of Other Intrinsic Targets

Nimodipine blocks the flux of extracellular calcium through L-type voltage-gated calcium channels. Voltage-dependent calcium channels (VDCCs) are widely distributed throughout the body and regulate the excitability and secretion in a diverse range of cell types. L-type calcium channels (LTCC) are expressed in the smooth muscles of vascular system on neurons and astrocytes [47,48]. 

In vivo applied nimodipine (0.1 µM) did not increase the recovery of dentate granule cells after 10 min of anoxia and did not reduce the decrease in ATP in dentate gyrus and CA1 [10]. Mechanisms behind neuroprotection for both calcium antagonists are not sufficiently clarified. Intravenous administration of nimodipine increased the firing rates of rabbit CA1 neurons, whereas nifedipine had no effect [21]. Conversely, nifedipine (1 µM) also reduced calcium spike potentials in young guinea pig CA1 [49] and rat CA3 neurons [50]. 

### 3.3. The Application Time of Nimodipine Is Crucial

Nimodipine was protective in OHSC when applied 4 h before damage or simultaneously to NMDA treatment, whereas 24 h preapplication of nimodipine or treatment 4 h after damage had no effect on neuronal damage. Preischemic application (1 h prior to ischemia) of nimodipine (0.1 or 0.3 mg/kg) was earlier reported to reduce the neuronal damage in the hippocampal CA1 subfield without affecting the postischemic local cerebral blood flow [35]. In that study, neuronal necrosis in the pyramidal cells of the hippocampus (CA3 and CA4 subfields) was only marginal and stayed unaffected from treatment with nimodipine [35]. In line with our findings, nimodipine-administered postischemic failed to preserve neurons from damage in a four-vessel occlusion model of global ischemia in rats [35]. These results show that nimodipine is able to protect neurons against ischemic damage if preincubated for a short time (1 h). Pretreatment with nimodipine before intracranial transection of the facial nerve led to an increased neuronal survival in the facial nucleus [51]. In addition, in mesencephalic neuron–glia cultures, pretreatment (30 min) with nimodipine (10 and 30 µM) reduced the degeneration of dopaminergic neurons after LPS (5 ng/mL) treatment [16]. Furthermore, nimodipine conferred neuroprotection in PC-12 cells only in a narrow therapeutic time window within the first 5 h [20]. In addition and in a model of intracranial facial nerve transection, nimodipine when administrated for 3 days preoperatively and 1 month postoperatively increased the number of surviving neurons [51]. Our results in OHSC support a concept of nimodipine-mediated protection in a temporally close vicinity to the injury. In our model, a 4 h pre-incubation or co-application with NMDA was followed by incubation with nimodipine until the fixation significantly reduced neuronal damage, but a pre-incubation 24 h before damage or application 4 h after starting the injury remained without any positive effects. Nimodipine might be qualified by these findings as a protective agent for elective neurosurgical procedures and should be considered in the planning of such interventions.

### 3.4. Effect of Nimodipine on Glia Cells

Among glia cells, microglia initiate the inflammatory response following various brain injuries, and once activated, migrate to the lesion site, proliferate and are the source of immunomodulatory molecules. Microglia-mediated attenuation in inflammation and oxidative stress is believed to protect neurons [19]. Nimodipine seem to act via multiple cellular targets. In the absence of microglia, nimodipine-mediated neuroprotection was abolished in dopaminergic neurons after damage [16] indicating direct effects on microglia. Nimodipine was also shown to block microglia phagocytosis but without interfering with inflammation or neuronal cell death mechanisms and was sufficient to enhance neuronal survival during inflammation [52]. However, nimodipine (5, 10, and 25 µM), when added in vitro to macrophages collected from splanchnic artery occlusion shock rats, significantly enhanced their phagocytic activity [53]. Nonetheless, microglia and macrophages differ in their inflammatory profile during injury as shown before [54]. 

The expression of LTCC, a target of nimodipine, was shown to be induced in activated microglia [55]. However, the effects on calcium household seem not to be responsible for nimodipine-mediated neuroprotection. Therefore, the possible role of microglia was investigated. Here, the effects of nimodipine and nifedipine on microglia viability and the number of IB4-positive cells after NMDA damage in OHSC were analysed. Both substances did not affect cell death of primary microglia or the number of microglia in OHSC in comparison to NMDA. In previous studies, the activation of microglia was inhibited after incubation with nimodipine, due to reduction in the production of nitric oxide (NO), tumour necrosis factor α (TNF α), interleukin-1ß and prostaglandin E2 from LPS-stimulated microglia [16]. Nimodipine was found to inhibit cell death triggered by amyloid ß in primary microglia and interleukin-1ß release from microglia (challenged with ATP 1 mM, LPS 1 µg/mL) [56]. Microglia cells were shown to express NMDA-receptors, but it is still a matter of debate if those are functional [19,57,58]. Microglia were shown to express NMDA receptors in the murine and human central nervous system, and these receptors triggered microglia activation in vitro and secretion of neurotoxic factors [58]. Conversely, pretreatment with NMDA antagonists did not affect production of NO or intracellular Ca^2+^ elevation induced by TNF, the mRNA expression of pro- or anti-inflammatory markers, or phagocytic activity of rodent microglial cells [57]. Further reports confirmed that microglial cells do not express functional NMDA in the rodent brain [19,59,60]. Taken together, it seems unlikely that stimulation with NMDA of primary microglia would change the effect on cellular death, since in OHSC after NMDA treatment, a massive increase in microglia numbers was observed [61,62].

In addition, nifedipine-mediated protection was associated with a reduction in proinflammatory cytokines from microglia in substantia nigra [36,63]. However, microglia pass through spatial, temporal, and functional diversity during homeostasis but also in diseases [64]. Possibly, microglial cells from various brain regions respond differently to nimodipine or nifedipine treatment. It is also plausible that nimodipine interact with microglia and change their function. In addition, direct interactions with neurons cannot be ruled out; however, blocking of calcium entry into neurons was observed by both nimodipine and nifedipine.

In human astrocytes, nimodipine significantly suppressed toxic secretions after treatment with interferon (IFN)-γ. Earlier results indicate that nimodipine-mediated protection involves microglia or/and astrocytes, since nimodipine improved neuroinflammation-induced memory deficits after systemic infusion of LPS [65]. Nimodipine, if applied before intracranial transection of the facial nerve, led to an increased amount of microglia, macrophages and activated astrocytes in the facial nucleus [51]. In agreement with this data, continuous nimodipine treatment led to higher glial fibrillary acid protein-immunoreactivity in astrocytes after resection of the facial and hypoglossal nerves [66]. Both studies indicate long-term effects of nimodipine on astrocytes and microglia. 

The modulation of neuroinflammatory responses due to LTCC in activated microglia and astrocytes needs further examination, and it is still not fully clear how nimodipine effects are mediated. However, this study showed that nimodipine seems to be a powerful agent which reduces excitotoxic damage and restricts spreading of secondary damage.

Few studies analysed the mechanisms of nimodipine actions in the CNS. After spinal cord injury, nimodipine-treated rats showed improvements in gliosis, CGRP+ fibre sprouting, and an increased KCC2 expression in lumbar motor neurons [67]. Furthermore, nimodipine downregulated lncRNA nuclear paraspeckle assembly transcript 1, upregulated miR-27a, downregulated microtubule associated protein tau, inhibited brain tissue cell apoptosis and enhanced brain cell activity, resulting in improved outcomes and cognitive performance [68]. The effects of nifedipine were not assessed in the mentioned studies. Nimodipine-mediated neuroprotection seems therefore to be a result of actions on glia cells and neurons.

## 4. Materials and Methods

All experiments involving animal material were performed in accordance with Directive 2010/63/EU of the European Parliament and the Council of the European Union (22 September 2010).

### 4.1. Primary Cell Cultures and Cell Lines

Primary microglia were detached from microglia–astrocyte co-cultures prepared from cerebral cortices of neonatal wild-type mice as described before (Grabiec et al., 2019). Brains were removed, and cells were dissociated after treatment with 4mg/mL trypsin (Merck Millipore, Burlington, MA, USA) and 0.5 mg/mL DNAse (Worthington, Bedford, MA, USA) in Hank’s balanced salts solution (Invitrogen, Carlsbad, CA, USA). This procedure resulted in the growth of a confluent astrocyte monolayer with attached microglia cells on top. Microglia cells were isolated from the monolayer by gentle shaking, and a purity of approximately 99% was reached.

Murine primary cells were cultured in medium consisting of DMEM (Invitrogen) with 10% FBS (Invitrogen) and 1 mL streptomycin/penicillin.

The microglial cells (5.000) were seeded into 24-well plates and treated with nimodipine (Bayer, Leverkusen, Germany, 0.1 µM, solved in ethanol), nifedipine (Bayer, 0.1 µM, solved in ethanol) or clodronate (10 µg/mL solved in water, Bayer for 24 h). For cell death analyses, propidium iodide (PI, 5 µg/mL, Sigma Aldrich, St. Louis, MO USA) was added 2 h before the fixation with 4% paraformaldehyde (PFA).

Cells were incubated with nucleic acid stain Sytox Green (Invitrogen, 1:10.000) and covered with DAKO fluorescent mounting medium (DAKO Diagnostika GmbH, Hamburg, Germany).

### 4.2. Organotypic Hippocampal Slice Cultures (OHSC)

**Figure 5:**
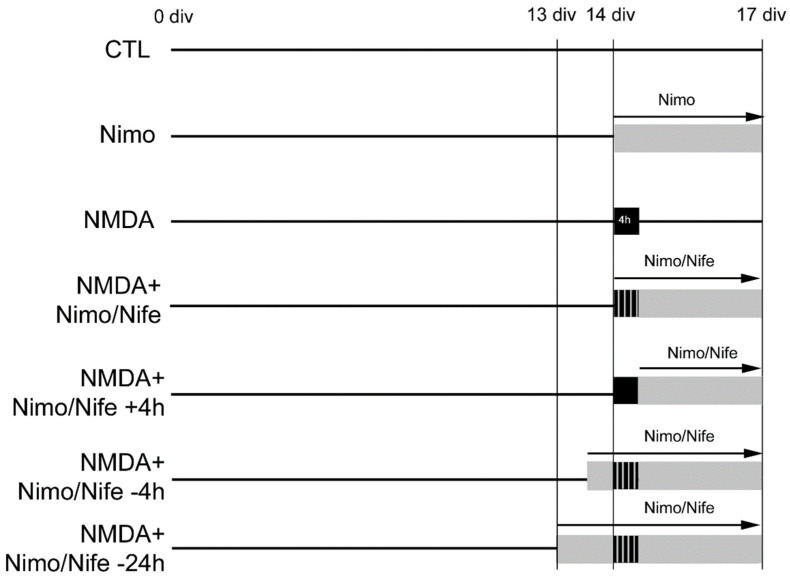
Treatment of murine OHSC. Murine OHSC were excitotoxically lesioned on 14 div. Next to the negative control (CTL) and positive control group damaged with NMDA (10 µM) for 4 h, one set of OHSC was treated simultaneously with NMDA (10 µM) and nimodipine (Nimo) or nifedipine (Nife). Further groups of OHSC were pretreated with nimodipine or nifedipine for 4 or 24 h before lesion.

OHSC were obtained from 5-day-old BL6J wild-type mice and prepared as published before [61,69,70,71]. After decapitation and dissection of the brains, the cerebellum and frontal pol were removed. Up to six 350 µM thin slices were obtained after cutting on vibratome VT 1200S (Leica, Wetzlar, Germany). The OHSC were incubated on inserts (Sarstedt, Nümbrecht, Germany) in culture medium (pH = 7.3) consisting of 47% MEM (Invitrogen, Carlsbad, CA, USA), 25% Hank’s balanced salt solution (HBSS), 25% normal horse serum (Invitrogen), 1% glutamine, 0,45% glucose (Braun, Melsungen, Germany), 1% penicillin/streptomycin (Invitrogen), and 0.8 µg/mL ascorbic acid (Invitrogen). The culture dishes were incubated at 35 °C in fully humidified atmosphere with 5% CO_2_. OHSC were divided into different experimental groups and treated with nimodipine or nifedipine in concentrations 0.1, 1 and 20 µM and NMDA (10 µM) (Figure 5). For detection of degenerating neuronal nuclei, 5 µg/mL PI was added 2 h prior to fixation. Afterwards, 4% PFA was applied for at least 24 h. For isolectin B4 (IB4, Vector laboratories, Burlingame, CA, USA) staining, OHSC were placed into a 24-well plate and washed with phosphate buffered saline (PBS) containing 0.03% (*v*/*v*) Triton X-100 (PBS-T) for 10 min. OHSC were then incubated with normal goat serum (diluted 1:20 in PBS-T) for 30 min and stained with FITC-conjugated IB4 diluted 1:50 in PBS-T containing 0.05% (*v*/*v*) bovine serum albumin (Sigma Aldrich) for 16 h. Thereafter, OHSC were washed with PBS/Triton for 10 min and then for 5 min with Aqua dest, and finally coverslipped with DAKO fluorescent mounting medium (DAKO Diagnostika GmbH, Hamburg, Germany). OHSC were analysed with Zeiss (LSM 700, Zeiss, Göttingen, Germany) and Leica (Leica DMi8, Leica, Wetzlar, Germany) confocal laser scanning microscopes. For detection of PI labelled degenerating neurons, monochromatic light at 543 nm and an emission bandpass filter of 585–615 nm was used. For visualization of IB4-labelled microglia, monochromatic light at 488 nm with a dichroic beam splitter (FT 488/543) and an emission band pass filter of 505–530 nm were used. PI and IB4-positive cells were counted in the granule cell layer (GCL) of the dentate gyrus (cells/GCL) using a MatLab script.

## 5. Conclusions

Since the excitotoxical damage was reduced after treatment with nimodipine, this substance might be a useful treatment option for a broad spectrum of neuronal injuries. Some aspects about nimodipine actions are crucial: first, the time of the application, and second, the concentration. Further, L-type channel blockers such as nifedipine are not protective in OHSC; for this reason, the mechanism of nimodipine seems to be unique.

## Data Availability

All datasets generated for this study are included in the article.
